# Updates to CDC’s Disability and Health Data System

**Published:** 2014-08-08

CDC’s Division of Human Development and Disability has recently updated its Disability and Health Data System (DHDS), a web-based data tool (available at http://dhds.cdc.gov) providing state-level data on the health of adults with disabilities in the United States.

Users can now view data in dual area profiles in addition to maps, data tables, and state profiles. Dual area profiles allow users to compare several pieces of health information for two geographic areas displayed side by side. In addition, 2012 Behavioral Risk Factor Surveillance System (BRFSS) data have been added to DHDS and are populated in maps, data tables, state profiles, and dual area profiles. Finally, interactive maps for mobile devices have been added so that users can view maps on a smartphone, tablet, and all web browsers except Internet Explorer 8 or earlier.

DHDS is intended to raise awareness of health disparities experienced by adults with disabilities in order to inform program and policy initiatives aimed at improving the health of adults with disabilities. Recent data on the health of adults with disabilities in specific states and health program areas are available through DHDS at http://dhds.cdc.gov. Questions regarding DHDS should be sent to dhds@cdc.gov.

